# User Experience Design for Online Sports Shoe Retail Platforms: An Empirical Analysis Based on Consumer Needs

**DOI:** 10.3390/bs15030311

**Published:** 2025-03-05

**Authors:** Yixin Zou, Chao Zhao, Peter Childs, Dingbang Luh, Xiaoying Tang

**Affiliations:** 1 School of Art and Design, Guangdong University of Technology, Guangzhou 510006, China; 2 Dyson School of Design Engineering, Imperial College London, London SW7 2AZ, UK; 3 Academy of Arts & Design, Tsinghua University, Beijing 100084, China

**Keywords:** experience design, online retail, shopping experience, e-commerce, sports shoe, LDA, Kano

## Abstract

Digital technologies represented by AR (Augmented Reality), VR (Virtual Reality), and digital twins, along with the expansion of metaverse platforms and digital marketing concepts, have attracted the attention of numerous sports fashion product consumers and brands, particularly in the category of sports shoes. Therefore, in the context of digital technologies, understanding the factors that affect consumer experience and the preferences in the online purchasing process of sports shoes is very important. This study employs Latent Dirichlet Allocation topic analysis to analyze 44,110 online user posts and comments from social platforms, extracting thematic elements of consumer experience needs for purchasing sports shoes online. The information obtained is further encoded and designed into a questionnaire, which is then utilized alongside the Kano model to analyze the overall preferences of consumer experience needs. The results indicate that webpage design and basic product information are considered as Must-be attributes for user experience needs; providing information on after-sales service policies and product comment, products’ special feature information, and online size testing are recognized as Performance attributes. Additionally, high-tech interaction methods, visual presentation, personalized customization, virtual try-on, apparel matching recommendations, and dressing scenario recommendations are identified as Attractive attributes. The study reveals that in the context of new digital technology development, the online shopping experience for sports shoes is enhanced across four dimensions: platform experience augmentation, product experience augmentation, user demand augmentation, and interactive experience augmentation. These four dimensions collectively constitute the holistic experience design for the online retail platform. Therefore, this research provides case references and theoretical insights for researchers and developers in the fields of brand marketing, experience design, and product service innovation.

## 1. Introduction

Driven by digital technologies such as AR (Augmented Reality) and VR (Virtual Reality), concepts such as the metaverse and digital twins are rapidly evolving and expanding. Such embodiments of the virtual world are gradually changing the way people socialize, entertain, and conduct business activities (Riva, 2023; Breugelmans et al., 2023). The diversity and interactivity brought about by technological innovation offer consumers an online shopping experience that is radically different from traditional e-commerce platforms (Ratchford et al., 2022). By 2024, the market size of the metaverse was expected to reach 1289.8 billion US dollars (Intelligence, 2021), fundamentally changing the way consumers interact with the digital world and reshaping the retail landscape (Sathyabama et al., 2024). In the retail sector, sports apparel as a combination of fashion and functionality has been one of the favorite product categories of consumers for several decades, constituting a significant portion of the retail industry; it is projected to generate approximately $109.35 billion in e-commerce revenue for the global sports and outdoor industry by 2027 (Statista, 2024). The sport shoe category is particularly noteworthy. Although these products are more commonly purchased in physical stores due to the need for consumers to physically interact with the items they intend to buy, sports shoe e-commerce still generates billions of dollars in revenue annually. Importantly, the market value of the sports shoe category is expected to continue to increase, with a compound annual growth rate (CAGR from 2019 to 2025) estimated at 10.4% (Grand view Research, 2019). If the online shopping experience for sports shoes can be enhanced within the metaverse environment, it could create even greater economic value. Current research on the retail experience of sports shoes tends to focus on the development of high-performance materials (Salopek Čubrić, 2021), marketing strategies (Slaton & Pookulangara, 2022), and studies on consumer preferences for the high-end sports shoes market (Raissa et al., 2024), with relatively little research conducted on consumer needs and real-time shopping experiences of online retail platforms. With the proliferation of online retailing platforms and the development and application of metaverse technologies, consumers’ shopping experiences and expectations for sports shoes are also continuously evolving. Therefore, this study aims to delve into the experience design of online sports shoe retailing in the current social context and technological state, and to conduct empirical analysis from the perspective of consumer needs. This study design considers the following research questions: RQ1: What are the feature words and related topics of discussion among the consumers regarding sports shoes online shopping? RQ2: What is the overall preference of consumers for the online shopping experience of sports shoes? RQ3: What aspects should be emphasized in the experience design of online retailing of sports shoes? In terms of research methodology and data analysis, this research has integrated several computational algorithms, including social network text mining and Latent Dirichlet Allocation topic analysis (hereinafter referred to as LDA topic analysis) to extract user-generated content, identifying high-frequency feature words. The application of this method primarily addresses RQ1. However, interpreting the overall preferences of consumers regarding the online shopping experience for sports shoes and determining the priority of their needs are separate issues that warrant further discussion. The Kano model, a classic framework for analyzing the relationship between consumer requirements and satisfaction, can be employed to address RQ2 and RQ3.

This research extracts topic-related keywords from authentic online posts and reviews regarding the online purchase experience of sports shoes using LDA topic analysis. By conducting a part-of-speech analysis of these keywords and reclassifying and decoding them, the underlying elements of user experience requirements are identified. To further explore consumer experiential needs, these thematic elements are structured into Kano survey questions. Combined with the Kano model and customer satisfaction indices, this approach facilitates the categorization of user needs and prioritizes the supply of various indicators. Ultimately, this study proposes case studies, strategies, and a framework for the experience design of online shopping platforms for sports shoes that target consumer needs.

The next section of the article reviews the relevant research literature. The third and fourth sections introduce the research methods, process, and data analysis, and presents the research results with illustrative examples, respectively. The fifth section discusses the results and proposes an innovation model for online retail experience. The sixth section summarizes the contributions and limitations of the study. This study investigates consumer behavior and preferences in the online shopping experience of sports shoes through empirical analysis. It contributes to deepening the understanding of consumer behavior patterns in the digital era and provides substantive suggestions for the design and operation of e-commerce platforms and provides a theoretical basis for the design innovation of sports shoes-related products and services.

## 2. Literature Review

### 2.1. Research on the Experience Design of Online Retail Platforms

**Figure 1:**
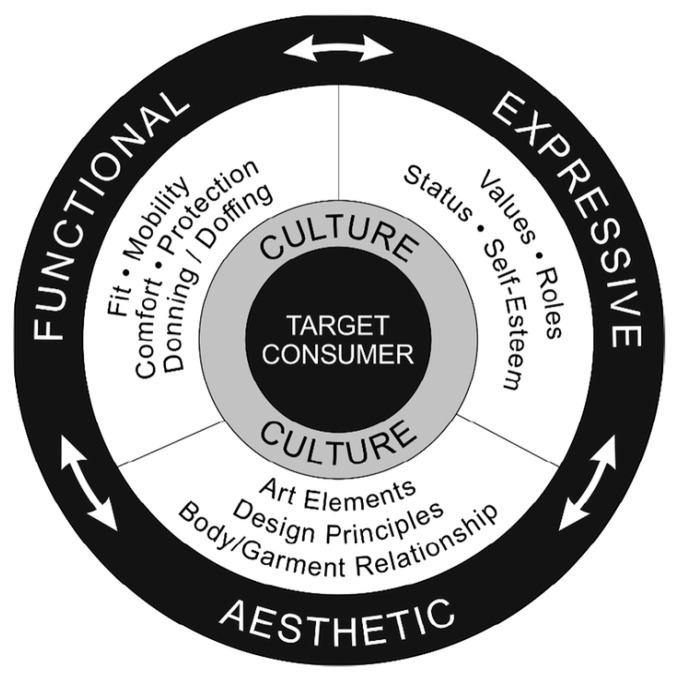
Functional, Expressive and Aesthetic (FEA) Consumer Needs Model (Lamb & Kallal, 1992).

From research on online retail experience, factors affecting consumers’ experience during the online shopping process that have been identified include brand image, visual page layout (Beig & Nika, 2022). Furthermore, some scholars have proposed that personalized services contribute to the improvement of consumer satisfaction and loyalty (Rane et al., 2023), an argument which is further developed by Sidaoui et al. (2020), stating that the content of related services should consider the subjective feelings and emotions of consumers during the shopping experience process. In addition to these perspectives, Mbete and Tanamal’s (2020) study emphasized that the key to creating a positive online shopping experience lies in informativeness, which pertains to the capacity to enlighten consumers regarding the characteristics of products. This insight underscores the significance of informativeness as a key determinant of customer satisfaction. Lamb and Kallal’s study in 1992 introduced the Functional, Expressive, and Aesthetic (FEA) Consumer Needs Model (Figure 1). This classic framework has been widely applied not only in the development process of apparel but also in reviewing whether the product information on online marketing interfaces meets consumers’ experiential needs.

The studies mentioned have provided a rich theoretical groundwork for this research. However, the development of digital technologies represented by the metaverse, and the extension of digital marketing concepts have had a profound impact on the fashion industry. An example of online marketing for sport fashion products is demonstrated by Nike selling sneakers in the metaverse (virtual space). Leveraging NFT (non-fungible token) technology, Nike established a store on Roblox, a widely popular online game with 50 million daily visitors, within a virtual sneaker marketplace. This initiative promotes the Nike brand in the virtual space and fosters relationships with young digital natives (Zou et al., 2022a). Anta, a worldwide sports apparel company, has integrated AI digital avatar technology with Style3D, empowering the transformation from 2D design drawings to 3D virtual clothing with flexible body simulation technology. This has enabled the creation of ultra-future sports virtual fashion, creating a new perspective and scenario as the virtual fashion show experience (Sohu, 2022). The implementation of AR for virtual try-ons has gained traction among major brands such as Gucci and Nike. AR try-ons allow customers to see how shoes look on their feet, providing a more interactive and engaging shopping experience (Ferrandez, 2022). In response to such phenomena, Casciani et al. (2022) propose that new technology-driven business models may shift from being exceptional cases to becoming a scalable market scope, thereby altering the processes of consumer behavior and consumption experience. This perspective was previously considered by McCarthy and Wright (2005), where they introduced the concept of technology as an experience, emphasizing that technology products are not just tools but also the mediums for experiences.

The use of emerging technologies in online marketing platforms is crucial for facilitating the shopping process for consumers and creating value (Saura et al., 2024). This view has been further advanced by other research, which considered the impact of new technologies on the marketing of physical and virtual stores. Such work asserts that the use of new technologies can provide a more convenient shopping experience, bring different products and services to consumers, improve shopping efficiency, and create a diverse online marketing environment, enhancing the interaction between consumers and brands (Abu-AlSondos et al., 2023). By presenting situational awareness and real-time performance data, it offers immersive online experiences (Bratu & Sabău, 2022), as well as engaging shopping scenarios (Tanveer et al., 2024). A good shopping experience will affect consumers’ decision-making process on social commerce platforms in terms of online trust, perceived risk, and purchase intentions (Lăzăroiu et al., 2020). In summary, the focus of online marketing research has shifted towards more comprehensive and multidimensional user experience design research. User experience design should focus on the emotional, cognitive, and sensory experiences of users when using technology products. The goal of user experience design is to achieve a “better user experience”, which includes not only visual and interface design but also functional design, information design, and other aspects (Berni & Borgianni, 2021). Norman’s and Ortony’s “Emotional Design” theory (Norman & Ortony, 2003), divides human needs into three levels: behavioral, visceral, and reflective. The interaction of these three levels contributes to the formation of a positive product experience. Zhao (2008) developed three meaning levels of the users’ needs model to demonstrate that the inter-relationships among the safety, utility, comfort, identity, emotion, and spirituality need categories constitute an interactive circularity with three meaning levels, such as the practical meaning, the social meaning, and the cultural meaning. With reference to these levels, the tangible and intangible product properties might involve economy, structure, function, technology, and esthetics to represent the users’ needs.

This study identifies a research gap concerning what factors influence consumers’ online shopping experiences in the current social and technological context, as well as what preferences consumers have for online shopping experiences. However, existing research on the online shopping experience for sports shoes is scarce, and a comprehensive view of the online retail experience is also lacking.

### 2.2. LDA Topic Analysis

The trajectory of research that focuses on online user-generated data has significantly broadened the scope of business research methodologies (Swider et al., 2024). This concept is further extended by the utilization of user-generated data (UGD), a term coined by Saura et al. (2021). UGD encompass all types of data and information that are individually produced by users through their interactions with digital marketplace components, such as behaviors, experiences, emotions, reviews, and comments (Saura, 2021). Within the realm of text mining of online user-generated data, Latent Dirichlet Allocation (LDA) topic analysis is a noteworthy method for text information mining and processing. The LDA model is an algorithm widely used for text topic analysis, capable of revealing hidden thematic structures within textual data, integrating a suite of analytical techniques such as word categorization, degree centrality, and frequency analysis (Feldman & Sanger, 2007) for document classification, information retrieval, and recommendation systems (Choi & Lee, 2020). The analysis enables keyword extraction, hot topic analysis, topic evolution, and thematic cluster analysis of the mined textual information (Zou et al., 2022a). LDA topic analysis is primarily utilized in the following areas: (1) Product Recommendation: Text mining of online reviews can reveal users’ emotional tendencies and interests. By analyzing the content of reviews, user interest sequences and communities can be constructed to achieve precise recommendations (Konstan & Riedl, 2012). (2) Product Comparison: By comparing reviews of competitive products, comparative opinion mining can be conducted to identify the competitive strengths and weaknesses of products. This aids in formulating effective marketing strategies (Kang et al., 2020). (3) Market Analysis: Text mining based on online reviews can be applied to sales forecasting, demand categorization, and brand evaluation (Zou et al., 2022a). These analyses can help businesses to understand market dynamics and consumer needs better. (4) Product Improvement: Evaluation of product attributes such as satisfaction, importance, and usefulness can be beneficial for identifying the opportunity value and direction for improvement in product attributes, thereby guiding product optimization (Kumar, 2022). Despite the numerous advantages and benefits of the LDA model, it has certain limitations when it comes to mining consumer requirements. For instance, the interpretability of topics can be poor, and words or phrases clustered under the same topic may lack semantic correlation, which makes the summarization of topic content less intuitive (Yu et al., 2023). To identify themes and the latent needs and priorities of consumers from a vast amount of textual data, a more systematic discussion that incorporates the Kano model is necessary.

### 2.3. Kano Model

**Figure 2:**
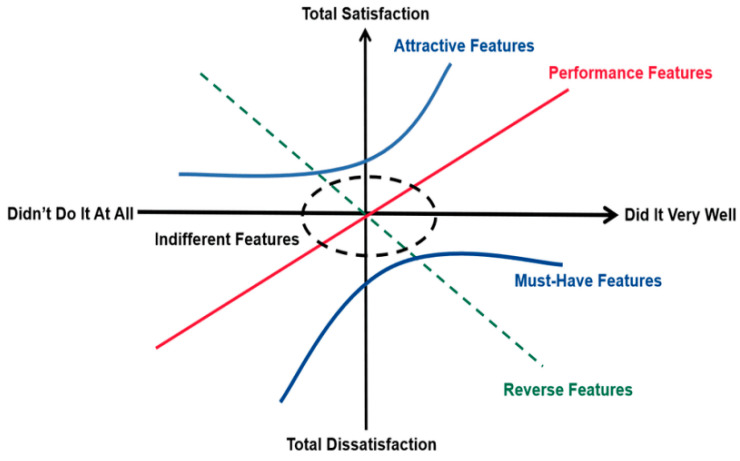
Kano model (picture from https://intl.finebi.com/blog/kano-model-analysis, accessed on 12 March 2024). Copyright © 2025 FanRuan Software Co., Ltd. All rights Reserved.

The Kano model is a classification tool used to understand customer needs for product or service attributes and reflects the relationship between different needs and customer satisfaction. This model was proposed by the Japanese scholar Noriaki Kano in 1984. The Kano model has attracted widespread attention among marketing practitioners and researchers and has developed into one of the most popular service quality models (Baier & Rese, 2020). The Kano model collects consumer needs and satisfaction with product attributes by issuing the Kano questionnaire. Based on the analysis of the questionnaire data, customer needs for product attributes are divided into five categories (shown in Figure 2): Must-be/Must-have, Attractive, Performance, Reverse and Indifferent (Kano & Mulavwa, 1984).

The Kano model is a classic framework for analyzing the relationship between consumer needs and satisfaction levels. Currently, the Kano model is widely applied in product development, service improvement, market research, and customer satisfaction measurement. (1) Product Development: The Kano model assists product teams in identifying which features are most likely to meet or even exceed customer expectations. By categorizing features according to the Kano model, teams can prioritize the development of those features that can significantly enhance customer satisfaction (Jin et al., 2022). (2) Service Improvement: In the service industry, the Kano model is used to identify and improve service attributes that have a significant impact on customer satisfaction. By analyzing customer satisfaction and expectations for service attributes, companies can more effectively enhance service quality (Sani & Febrian, 2023). (3) Market Research: The Kano model is utilized in market research to analyze customer needs and preferences. Through surveys and data analysis, researchers can understand customer expectations and satisfaction with different product attributes, thereby providing a basis for market strategies (Andriani et al., 2021). (4) Customer Satisfaction Measurement: The Kano model offers a method for measuring customer satisfaction. By analyzing customers’ emotional responses to product or service attributes, businesses can more accurately assess customer satisfaction and make improvements accordingly (Kohli & Singh, 2021).

Building on the research questions previously stated, the key to this study lies in analyzing the demands of consumers regarding the online shopping experience for sports shoes and the prioritization of various experiential attribute requirements. It should be noted that current research utilizing the Kano model is largely based on data from Kano questionnaires, which have certain limitations in terms of sample coverage and representativeness. The problem setting requires pre-known characteristics, necessitating various market and customer analyses in advance, such as competitive analysis and customer interviews, to identify potential features (Chapman & Callegaro, 2022) and avoiding issues such as overly subjective problem settings (Mikulić & Prebežac, 2011). User-generated information, represented by online posts and reviews, is an effective channel for consumers to directly express their opinions on specific events or topics. The emergence of big data from online reviews provides an effective path to address the limitations of traditional Kano questionnaire setting. As online reviews represent the genuine expressions of users regarding their experiences with products, the consumer needs information derived from these expressions is also more authentic.

This paper proposes a method for identifying consumer needs based on online reviews, which employs LDA topic analysis for thematic analysis of online comments and generates meta-themes to guide the design of the Kano model questionnaire. Secondly, by applying the Kano model to analyze the requirements, the analysis results can be used to identify the core needs of users and overall preference of consumers for the online shopping experience of sports shoes.

## 3. Research Process and Results

### 3.1. LDA Analysis

**Figure 3:**
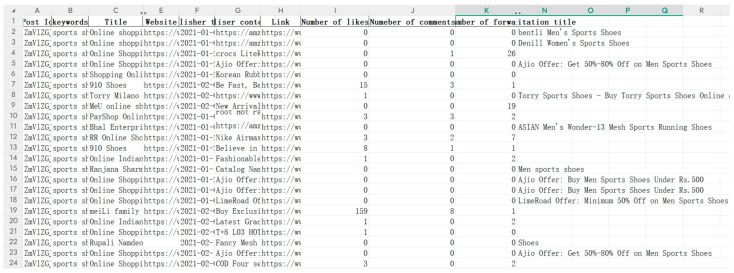
Collected post content.

This study used Python 3.8 to analyze posts (containing images, videos, or hyperlinks to other websites) and comments about online shopping for sports shoes on five major social networking platforms, Twitter, Facebook, Redbook, Tiktok, and Zhihu. The keywords set were “sports shoes online shopping, sneaker online shopping, sports shoes/sneaker online shopping experience, sports shoes/sneaker online shopping platform interface design, sports shoes/sneaker online shopping platform interaction design, sports shoes/sneaker online sales interface design”. Following completion of initial data collection, further data processing operations were undertaken including filtering, adding, deleting, modifying and reorganizing, and invalid and erroneous data were removed. The specific steps include the following: 1. Removal of special characters: Eliminate unnecessary symbols and characters from the data, including punctuation, symbols, and HTML tags, to ensure that the text data are in plain text. 2. Removal of stop words: Remove unnecessary words and stop words, such as “a” and “the”, which do not contribute to text analysis. 3. Standardization of words: Normalize the vocabulary in the text data, such as unifying different capitalizations, tenses, or forms of the same word into a consistent format. 4. Stemming and lemmatization: Perform stemming and lemmatization on the vocabulary in the text data to reduce redundancy and make them more efficient. 5. Spell check: Conduct a spell check operation to identify and correct spelling errors present in the data (specific operational steps are presented in the attachment). This process retained 20,017 post data and 24,093 comment data related to the topic, totaling 44,110 data samples. Subsequently, LDA topic analysis was applied for detailed thematic mining. A portion of the collected metadata were sorted as shown in Figure 3 (due to the sheer volume of data, only selected data examples are shown in the paper).

**Figure 4:**
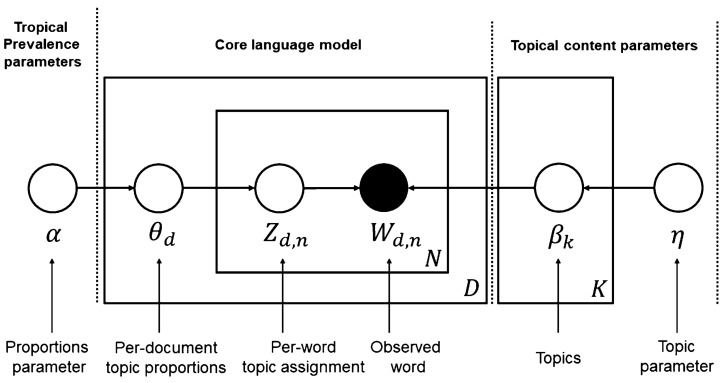
The LDA model (Blei, 2012, p. 81). Copyright © 2025 ACM, Inc.

Drawing from Bayesian principles, Blei introduced the Latent Dirichlet Allocation (LDA) model in 2003. As depicted in Figure 4, Blei detailed the LDA algorithm in 2012, illustrating it with “plates” represented by square boxes. Here, “N” denotes the ensemble of words in an individual document, “D” signifies the entire set of documents, and “K” refers to the various topics under consideration. The circular nodes indicate the probabilistic parameters. Specifically, “W_d,n_” is recognized as an observable word within a document. In contrast, the topics themselves, their distributions, and the assignments to documents remain latent (Kim et al., 2022).

**Figure 5:**
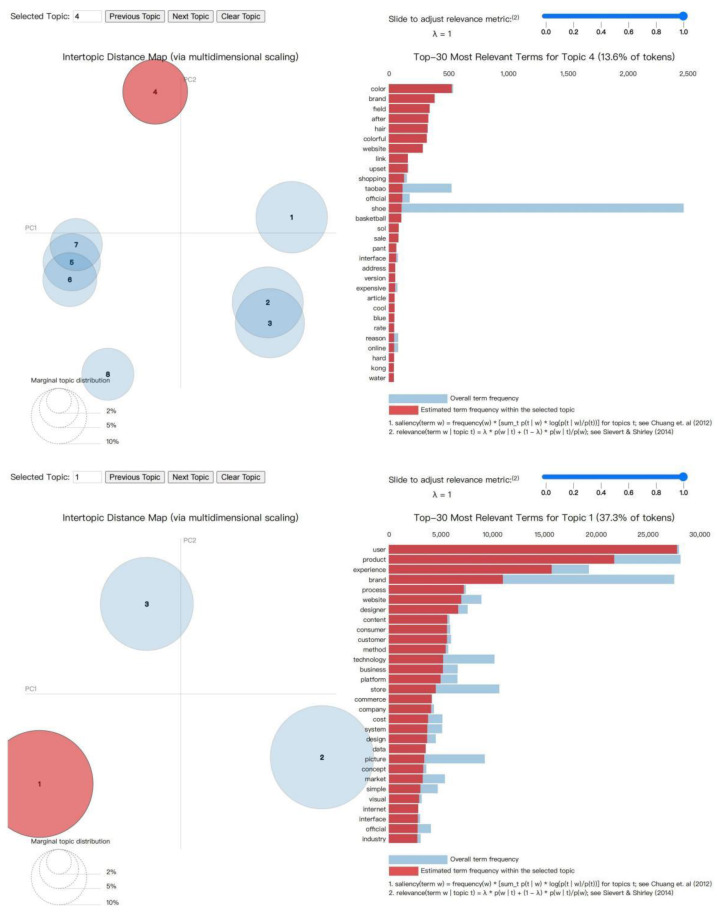
LDA topic distribution visualization results (Chuang et al., 2012; Sievert & Shirley, 2014).

Default system comments, comments with insufficient information, and repetitive comments were excluded to ensure data quality. As already indicated after a cleaning process, 44,110 data records were obtained. By analyzing the content of the comments, word segmentation, topic identification, and visualization were concluded. This led to the creation of two major thematic visualization maps. Figure 5 displays a visualization of the results of the thematic analysis of posts made by online users regarding key terms, which represents the evaluative data from the users. In the upper right corner of the figure, parameter data can be observed; a higher value of λ indicates that words with higher frequencies are more relevant to the theme, and a lower value of λ suggests that more unique and specific words are relevant to the theme. The study set λ = 1, selecting the words with high frequency in each category as the outcome of topic clustering. On the left side of the figure is a view of the topic distribution; on the right is the “Term Bar Chart”, which shows the top 30 most probable characteristic words for each theme (Vukanti & Jose, 2021).

From the topic distribution view, there is no overlap between the bubbles, which means that the similarity between each topic is small, indicating that this topic modeling of the online sales experience of sports shoes is effective, and the data obtained are suitable for further analysis and research. There are three large bubbles in the topic distribution map about posts, which means that three major categories of topics are generated, and there are 11 bubbles in the topic distribution map about comments, i.e., 11 major topics and 220 feature words are generated. Table 1 shows examples of related feature words. The utilization of the LDA topic analysis method involves clustering to extract feature words from online review corpora, which may result in themes that are not distinctly defined, and potential overlaps between themes. For instance, the same term may appear in multiple themes, leading to a deficiency in the independence and orthogonality of the themes. To obtain accurate user requirement information, this study involved combining the authors’ knowledge of marketing and experience and utilizing methods such as local contextual co-occurrence of words and expansion of short texts to judge parts of speech (for example, nouns may represent product attributes, while adjectives may reflect users’ emotional attitudes towards these attributes, etc., and some meaningless features were not included in the coding). The topics were named based on the attributes of the feature words (shown in the second column “Topic” in Table 1).

**Figure 6:**
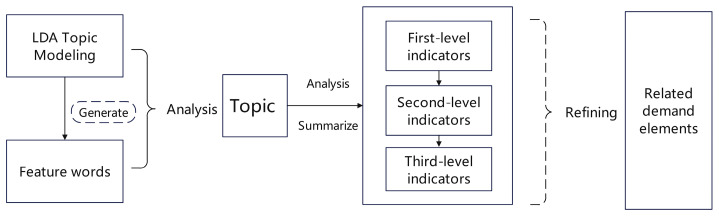
Transforming LDA topic modeling into a three-level indicator of user needs.

Furthermore, the effective transformation of superficial part-of-speech features into deeper user needs is a crucial process for conducting management decision optimization research. After conducting a thematic clustering analysis of online posts, this study extracted the key components of the online shopping experience for sports shoes, namely all the feature words and the major thematic categories they represent. However, to further organize user experience needs, more systematic information needs to be extracted. Therefore, based on the thematic analysis, three levels of detailed factors were constructed through the interpretation of word meanings, and then, an overall user demand framework was proposed (Figure 6).

Among them, the first-level indicators remain unchanged, that is, the 11 major topic categories generated by the LDA topic analysis. The third-level indicators are the feature words that appear in the LDA topic analysis. The second-level indicators are the result of secondary coding based on the word meanings of the third-level indicator feature words. The coding process mainly relies on the part of speech of the feature words and the topic they are in for refinement. As an example, under the topic of “platform and webpage”, the feature words of “term, item, guide, channel” can be decoded as “webpage information and layout”, etc. The detailed different level indicators are coded and categorized in Table 2.

Within these 12 themes, an in-depth analysis was conducted for relevant content. The category of Product Information contains a multitude of feature words with substantial information, including “products categories, basic information, logo, material /fabric, price, color, details, shape, product positioning/design positioning, product features/advantages, design process, making process, source, product data, product usage data, product quality”. This indicates that when consumers purchase sports shoes, they not only care about the basic information of the product (e.g., price, color, and shape), but also have a strong interest in more detailed characteristics (e.g., performance, quality, material, and functionality), and product performance is directly related to the description of the application scenarios. Detailed product information can significantly influence consumer behavior and decision-making, increase perceived value and consumer satisfaction, and influence purchase intentions (Rosário & Raimundo, 2021). For brands, in a competitive market environment, each brand needs to differentiate themselves by providing detailed product information to demonstrate their unique advantages, which is essential for creating a compelling online shopping experience and helps attract potential consumers.

The second major theme, Service, includes “service items, shopping process, appointment, delivery, after-sale, attitude”. This indicates that besides products, services are also significant considerations that consumers discuss and emphasize in the process of online shopping. In online shopping, consumers are unable to directly touch and try on products, so they rely more on service and experience. Quality service (e.g., detailed product descriptions, fast delivery, good after-sales service) can increase consumers’ trust and sense of security, and reduce the uncertainty caused by information asymmetry (Zavolokina et al., 2021). In addition to basic service items, there are also topics such as “interactive mode, display mode, sports categories, events, dressing scenario, dressing time, relevant clothes & matching”, that are, possibly surprisingly, also commonly discussed by consumers in addition to the basic products and services. Here, these are classified as “Additional Services”, which provides a lot of information for further in-depth discussion in later research. It is very important for brands to create more unique shopping experiences for consumers through additional services and good products, to help win market share in fierce competition. Another finding is that there are many positive and negative feature words, which further supports the idea that a good online shopping experience is very important for a brand or a company to enhance consumer satisfaction and enjoyment, develop consumer loyalty, and promote long-term relationship maintenance.

### 3.2. User Demands Classification Based on the Kano Model

(1) Questionnaire question setting

Integrating the LDA topic analysis, a basic construction of the elements constituting users’ online shopping needs was established. However, to ensure the focus of the research questions and to avoid the issue of an overly large sample size in the questionnaire, themes were selected related to the online sales experience from the analysis results mentioned. Generally, the experience design of online shopping includes the following: online shopping interface design, optimization of the shopping process, interactive recommendation system, strong interactive function design, efficient service, and information provided by the platform, etc. Therefore, this study selected related content from the previous three-level-indicator framework to generate Kano questionnaire questions in order to obtain more accurate user experience needs (the selected experience elements are highlighted with shading in Table 3).

Indicators related to online shopping experience were selected from Table 3. As for first-level indicators, these include “Platform and webpage, Brand, Product information, Services, Additional services, Stakeholders, Lifestyle, Community, Feedback of product or brand” are shown in Table 4 as “Themes”. The selected second-level indicators (shown in Table 3 highlighted with shading) which related to experience design under the list were transferred as Topic in Table 4. “Interactive display methods” was added in Table 4 as one of the themes, as a way to display the product information and as a new technique to integrate with platform and webpage design. These indicators were further developed into more topics with assigned numbers, along with “detailed descriptions” of these topics. These “specific descriptions” serve as the basis for the design of questionnaire items and as detailed descriptions of the survey questions. The Kano questionnaire is shown in Table 4.

Based on the Kano model for customer requirement classification, the specific details of the option design in this research are as follows: Firstly, when using questionnaires for research, questions regarding the same feature/service are asked from both positive and negative perspectives to acquire users’ attitudes and feelings towards the provision or absence of the element. The positive question refers to “If there is a feature/service, what is the evaluation?” The negative question refers to “If there is no feature/service, what is the evaluation?” For each question, responses are given as five options: “Highly Like”, “As Expected”, “Indifferent”, “Acceptable”, and “Highly Dislike” (as shown in Table 5). Secondly: The cross-tabulation of the options between the positive and negative questions yields six types of attributes; the Attractive attribute (A) refers to features/services that exceed customer expectations; the Performance attribute (O) indicates that having a certain feature/service will increase satisfaction, while its absence will decrease it; the Must-be attribute (M) indicates that having a certain feature/service does not increase satisfaction, but its absence will decrease it; the Indifferent attribute (I) means that having or not having a certain feature/service does not affect satisfaction; the Reverse attribute (R) indicates that the absence of a certain feature/service would lead to higher satisfaction; and the Questionable attribute (Q) suggests that the user did not fully understand a question or provided an incorrect answer (Kermanshachi et al., 2022).

(2) Questionnaire survey process

The survey was conducted from 6 April to 10 September 2024. This questionnaire is divided into three parts: The first part consists of basic user information, including user age (single-choice question), gender (single-choice question), occupation (single-choice question), educational level (single-choice question) and websites or apps used for online purchases of sports shoes (multiple-choice question). The second part is the Kano questionnaire, which investigates 30 demand elements (specific questions are shown in Table 4). Each element is composed of a pair of positive and negative questions. For example.

Positive question: If graphical presentation was used to provide sports shoes basic information (including color, price, style, etc.) would you be satisfied with it?

Negative question: If graphical presentation was not used to provide sports shoes basic information (including color, price, style, etc.) would you be satisfied with it?

Answer options: Highly Like, Acceptable, Indifferent, As Expected, Highly Dislike.

(3) Recruitment of Participants

Online Recruitment: Participants were recruited online through the dissemination of questionnaire links via social media platforms (such as WeChat, Weibo, Instagram, Facebook) and e-commerce platforms. Offline Recruitment: Offline recruitment was conducted by distributing QR codes for the questionnaire at selected sports brand stores and fitness gyms in several cities. Incentive Measures: Participants who completed the questionnaire were given the opportunity to enter a lottery for small gifts.

Participants were recruited through the “Questionnaire Star” platform, which allows researchers to reach users via multiple online channels, including social media platforms, email distribution, and online communities of sports shoe enthusiasts. To ensure relevance, screening questions confirmed participants had purchased sports shoes online in the past year. The proportions of the male and female groups were 50.19% and 49.81%, respectively. In terms of age groups, the 18–25 age group accounted for 18.16%, the 26–30 age group accounted for 23.98%, the 31–40 age group accounted for 31.97%, the 41–50 age group accounted for 18.64%, the 51–60 age group accounted for 6.32%, and those over 60 years old accounted for 0.93%. In terms of occupation, design-background bachelor students accounted for 19%, design-background master students accounted for 8%: design-background Ph.D. candidates accounted for 5.8%, design-background teachers accounted for 11%, designers accounted for 10.2%, company managers 5%, self-employed 14%, other 20%, and marketing workers 7%. There were 52% participants from urban, 32% from suburban, and 16% from rural environments. A total of 2157 questionnaires were collected, of which 2122 were valid, yielding an effective response rate of 98.4%. The responses reflect different domains of design, marketing, experience, brand, and management, focusing on consumers of sports shoes.

(4) Reliability and validity test

The questionnaire was distributed exclusively online, leveraging the broad reach of digital platforms. The use of an electronic format ensured convenience for participants while allowing for efficient data collection and automated processing.

The reliability and validity of the questionnaire were tested using SPSS software version number 26.

Reliability: Cronbach’s Alpha was used to assess internal consistency, yielding a value of 0.89, indicating high reliability.

Validity: Factor analysis confirmed the questionnaire’s construct validity, with all items loading significantly on their intended factors.

(5) Statistical results of this questionnaire survey

Table 6 displays the statistical results of the Kano survey. The Kano model results show the proportion of the six qualities where the columns A, O, M, I, R, Q represent. Ultimately, the Kano classification rule considers the category with the maximum frequency value as the quality’s class, which is indicated by column 1 in Table 6. The Better and Worse value conditions: Both Better (satisfaction impact) and Worse (dissatisfaction impact) are used to determine the sensitivity of users to changes in the level of features/services. Better (satisfaction impact) = (A + O)/(A + O + M + I), and this indicator ranges between 0 and 1; the larger the value, the greater the sensitivity, and the higher the priority. Worse (dissatisfaction impact) = −1 * (O + M)/(A + O + M + I), and this indicator ranges between −1 and 0; the smaller the value, the greater the sensitivity, and the higher the priority (Mikulić & Prebežac, 2011).

**Figure 7:**
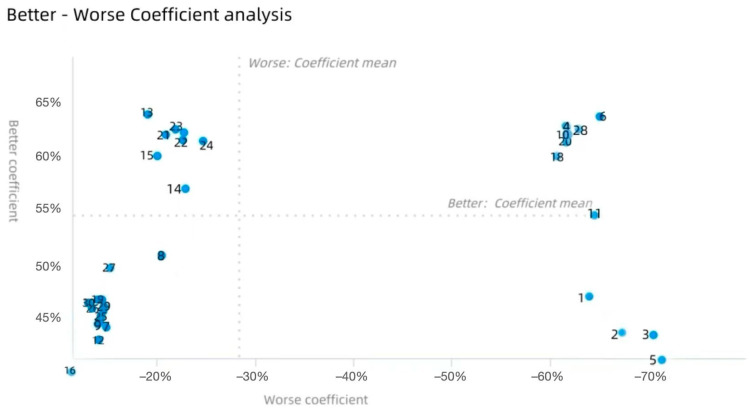
Better–Worse scatter plot (blue spots represent the positions of different topics in the scatter plot).

According to the research findings, a Better–Worse scatter plot was constructed (as shown in Figure 7), with the Better value as the horizontal axis and the Worse value as the vertical axis, and the average value of better and worse as the origin intersection. In the context of the Better coefficient (represented on the vertical axis), a positive value indicates that the presence of a feature enhances user satisfaction, whereas a negative value suggests that the presence of the feature diminishes user satisfaction. The Worse coefficient, displayed on the horizontal axis, features negative values that signify the absence of a feature increases user dissatisfaction. The greater the absolute value, the more significant the impact of the feature’s absence on user dissatisfaction. The quadrant in the upper right represents a Performance attribute, including Topic No. 4, No. 6, No. 10, No. 11, No. 18, No. 20, No. 28. The lower right quadrant represents a Must-be attribute, including topic No. 1, No. 2, No. 3, No. 5; the upper left quadrant represents an Attractive attribute, including topic No. 13, No. 15, No. 17, No. 21, No. 22, No. 23, No. 24. The lower left quadrant represents an Indifferent attribute, including topic No. 7, No. 8, No. 9, No. 12, No. 14, No. 16, No. 19, No. 25, No. 26, No. 27, No. 29, No. 30.

## 4. Result Statistics and Findings

From the research findings, the preliminary data analysis results indicate that the current consumer responses are primarily composed of four attributes: M, I, A, and O. Since the I attribute has minimal impact on consumer satisfaction, only the results for the M, A, and O attributes are compiled here for further detailed analysis and summary shown in Table 7, where the first column represents the Kano attribute category, the second column represents the overarching theme, the third column represents the specific topic, and the fourth column reveals the implicit characteristics of experience design and consumer needs associated with the topic.

### 4.1. Fundamental Demands

The topics selected as Must-be attributes and their corresponding numbers are No. 1 Beautiful interface, No. 2 Smooth operation and No. 3 Clear layout. These topics belong to the broader theme of “webpage design”. In the context of the rapid development of e-commerce, webpage design is an important factor influencing online shopping behavior. An interface that is easy to navigate and visually appealing can reduce the user’s cognitive load, improve shopping efficiency, and enhance the sense of pleasure, directly affecting the overall shopping experience (Shang et al., 2020). This is one of the essential aspects that brands or businesses must prioritize. No. 5 Product basic information belongs to the broader theme of “product information”. Echoing the viewpoints presented by researchers in the previous text, consumers have diverse needs for product information during the purchasing process, including price, specifications and functions. Providing comprehensive product information can meet the varied needs of consumers, enabling them to compare and choose based on their specific requirements (Tzeng et al., 2021).

In addition to these fundamental requirements regarding interface design and product information, it is noteworthy that No. 4 Immediate information and No. 6 Product special features information are selected as Performance attributes. This phenomenon indicates that current online sports shoe sales platforms have issues such as untimely updates of product information and inconspicuous product feature details. It also reflects consumers’ demand for personalized and value-added products. Providing distinctive information about sports shoes on online sales platforms will assist merchants in standing out in fierce market competition characterized by homogeneity, thereby satisfying consumers’ personalized needs and preferences and enhancing the shopping experience and satisfaction.

### 4.2. The Enhancement of Service Assurance and Security Needs

The results of the Kano analysis indicate that topic No. 18 After-sales service policy is a Performance attribute. Researchers conducting further investigations have found that some online sports shoe shopping platforms indeed do not provide such information. Offering clear after-sales service policies can increase consumers’ trust and sense of security in online shopping platforms. A well-defined after-sales service policy can help reduce return rates and consumer disputes. When consumers understand policies regarding returns, exchanges, and repairs before making a purchase, it can minimize misunderstandings and dissatisfaction caused by unclear policies, thereby reducing return rates and enhancing the shopping experience (Shokouhyar et al., 2020). Another Performance attribute topic is No. 28 Product comment. This research found that while some online sales platforms offer this type of feedback information, others do not. From the perspective of consumer experience, most consumers want this functionality improved, which also reflects, to some extent, the lack of trust consumers have in products or brands during the online shopping process. When making purchasing decisions, consumers rely on the opinions and experiences of others. Customer comments can serve as a third-party information source, offering genuine feedback on products and helping consumers establish trust in the products and platforms (Fu et al., 2020). However, for businesses, the quantity and quality of online customer reviews can impact the sales volume of products. Positive reviews can enhance consumers’ willingness to purchase (Zhu et al., 2020). If the provision of a “comment” section is increased in retail platforms, it serves not only as a tool for information communication with consumers but also as an important channel for businesses to obtain feedback. However, it is acknowledged that there is a risk of the comments containing false or misleading information, particularly in the form of negative comments about the product. To mitigate this risk, platforms should implement robust moderation mechanisms to filter out false or malicious comments while still allowing genuine consumer feedback to be shared. Additionally, businesses can leverage data analytics and sentiment analysis tools to identify and address genuine concerns raised by consumers. Overall, while there are potential challenges, the benefits of having a comment section as a form of supervision to improve and enhance product and service levels outweigh the risks when managed effectively.

No. 20 Size testing is selected by the audience as a Performance attribute as well. For sports shoes, a significant difference between online and physical shopping is the inability to try on sizes; incorrect sizing is one of the main reasons for sports shoe returns (Hajjar et al., 2021). Online shoe-size testing technology primarily relies on high-tech methods such as 3D vision, computer vision, artificial intelligence, and machine learning. These technologies can recommend the appropriate shoe size by analyzing consumers’ foot shape data. Currently promoted cases in the market include Nike’s announcement of the “personal shoe size measurement expert”, the mobile version of Nike Fit, which has been launched on its official Nike App. Utilizing Apple’s LiDAR laser radar technology, it provides precise shoe-size recommendations by scanning consumers’ feet. Volumental’s FitTech^TM^ technology platform provides personalized footwear recommendations for consumers through 3D foot scanning and big data technology. Additionally, there are applications such as E-Size, which use computer vision and artificial intelligence algorithms to determine the linear parameters of the foot. However, further market penetration is yet to occur.

### 4.3. The Enhancement of Technological, Interactive and Immersive Needs

The topics selected as Performance attributes also include No. 10 Interactive display of comprehensive product features and No. 11 Interactive display of making techniques. To achieve an interactive presentation of this information, relevant interactive technologies or media are required to execute it. For display methods, No. 13 VR and AR, No. 15 Video and No. 17 Touch were selected as Attractive attributes. These technologies or media for information presentation are important elements that consumers expect in their online shopping experience. In experience design, the application of such technologies has the following advantages: (1) Enhancing the shopping experience. Against the background of a metaverse retail environment, technologies such as VR, AR, video and other techniques provide an immersive shopping experience, enabling consumers to experience products more intuitively in an online environment. This experience transcends traditional two-dimensional images and textual descriptions, offering consumers a richer and more realistic way to understand products (Chen & Mu, 2024). In addition, the data from this study indicate that for the sports shoe category, when promoting high-performance features such as waterproofing, breathability, high elasticity, and cushioning to consumers, the communication effect may be insufficient without visual aids due to the high levels of professional knowledge involved. Videos, as a more intuitive form of visual communication, can help consumers quickly grasp more product information. (2) Improving decision-making efficiency. By using VR and AR technology, consumers can more accurately predict the size and appearance of shoes, thus reducing this risk. Such visual tools can help consumers quickly obtain key product information, such as functionality, design details, etc. This helps consumers in making purchasing decisions more swiftly (Lixăndroiu et al., 2021). (3) Enhancing brand competitive advantage. As an increasing number of online platforms adopt new technologies, offering VR, AR and new media content has become a way for branding. These technologies not only improve user experience but also serve as a hallmark of brand innovation and leadership position (Baytar et al., 2020). (4) Increasing social exposure. Modern consumers enjoy sharing their shopping experiences on social media. The use of digital technologies can provide more interesting and shareable content, increasing the product’s social exposure. In the online shopping environment, the implementation of tactile experiences poses a challenge, as consumers cannot directly interact with products as they would in a physical store. However, with technological advancements, some methods have been explored and implemented to simulate haptic feedback. Three-dimensional vision and haptic technology: For example, using TanvasTouch technology, dynamic tactile surface effects can be created on screens, allowing users to feel different textures and qualities when browsing fashion products (Ornati & Cantoni, 2020). Multimodal Feedback: Combining various haptic feedback mechanisms, such as mechanical vibrations, thermal stimulation, and electrical stimulation, to provide a richer tactile experience (Huang et al., 2022). Skin-integrated haptic interfaces: Developing haptic interfaces that can be integrated into the skin, which can provide immersive tactile feedback in virtual and AR (Keef et al., 2020). These methods can simulate various tactile sensations, thereby providing a more authentic online shopping experience. However, it is acknowledged that implementing such advanced haptic feedback mechanisms in the context of home-based e-shopping may face challenges, particularly in terms of accessibility and user adoption (Shi et al., 2023). To address these challenges, several strategies can be employed. Incremental Adoption: Start by integrating simpler haptic feedback mechanisms, such as vibration-based feedback, which are already widely used in mobile devices and can be easily incorporated into existing e-commerce platforms. This can help familiarize users with the concept of haptic feedback in online shopping. Collaboration with Device Manufacturers: Work closely with manufacturers of wearable and haptic-enabled devices to ensure compatibility and seamless integration with e-commerce platforms. This can help in providing a consistent and engaging user experience across different devices. Gradual Integration: Introduce haptic feedback features gradually, starting with popular product categories such as fashion and electronics, where tactile feedback can have the most significant impact on user decision-making. By addressing these challenges through a phased approach and leveraging existing technologies, it is possible to enhance the online shopping experience with haptic feedback, even in a home-based e-shopping context.

**Figure 8:**
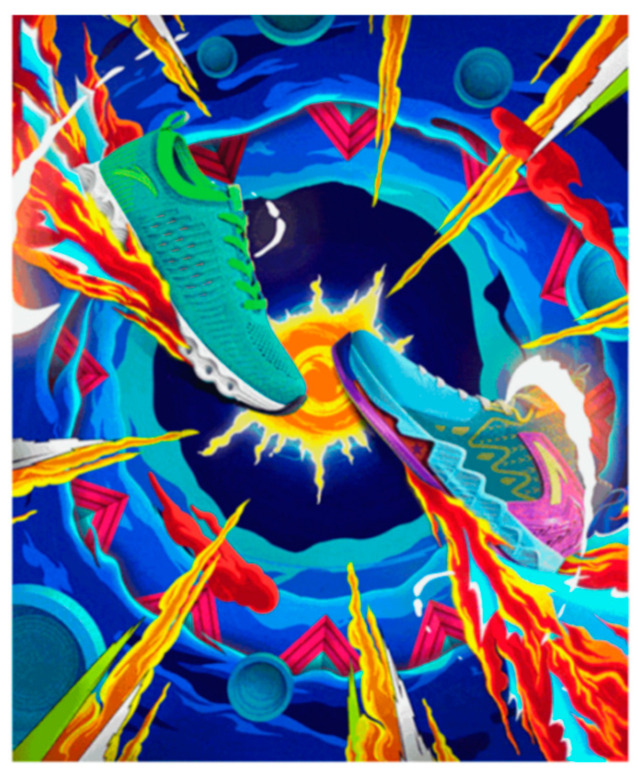
ANTANUI (Anta, 2024). Copyright © 2012–2025 by www.ANTA.cn (accessed on 23 February 2025).

Topics selected as an Attractive attribute include No. 21 Personalized customization and No. 22 Virtual try-on. Inextricably linked to the previous “Interaction methods”, the online presentation of sports shoes can be enhanced through AR and VR technologies, allowing consumers to view the shoes in three-dimensional space and even try them on virtually, showcasing the actual wearing effect and usage scenarios of the shoes, thereby improving the immersive shopping experience. Regarding these two topics, there are some relevant market application examples. Anta has launched the personalized product customization service system “ANTANUI” (shown in Figure 8), where consumers can freely customize their own shoes by choosing different shoe shapes, colors, and patterns on the online platform. The launch of this online business has helped the Anta brand expand into new niche markets and demonstrated the brand value and attitude of embracing emerging technologies (Anta, 2024).

**Figure 9:**
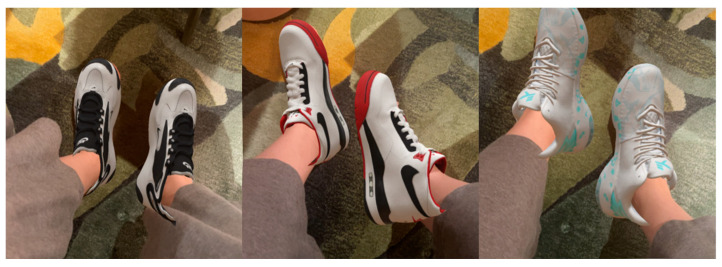
AR try-on. (The product was tested by the principal author and the images generated on the Dewu app platform, (version 5.62.1)). Copyright ©2016–2024 by Shanghai Dewu Information Group Co., Ltd.

As a further example, the Chinese online shopping app named Dewu has won the “Golden Trend Award” for its annual empowerment innovation award based on the application of features such as “AR try-on”. The technical team has achieved a “pixel-level” representation of the 3D models of the products. When users try on the products, the model can adapt to different foot angles as well as the movement and rotation of the feet, thus achieving a very realistic online try-on effect (shown in Figure 9), which has attracted many young consumers.

AR and VR technologies enable personalized shopping experiences, allowing consumers to adjust product options according to their preferences, such as color and size, which is difficult to achieve in traditional online shopping. For example, lightweight VR solutions, such as mobile phone-based VR experiences, have emerged as cost-effective alternatives for consumers. Numerous fashion brands and e-commerce platforms have begun implementing virtual fitting room technologies, enabling consumers to utilize virtual mirrors to simulate garment try-on effects from home. For instance, Figure 9 demonstrates a virtual try-on test conducted by the author through an app. With technological advancements and the maturation of the industry chain, VR equipment costs are progressively decreasing. Through cost reduction strategies, enhanced user experience optimization, and phased implementation approaches, these technologies are anticipated to evolve into critical factors for enhancing brand market share in the foreseeable future.

### 4.4. Emotional Needs Enhancement: Personalization, Socialization, Sense of Belonging

Topics selected as Attractive attributes are No. 23 Dressing way recommendations and No. 24 Dressing scenario recommendations, which fall under the category of “additional services”. Based on the results obtained from the LDA topic analysis mentioned earlier, keywords related to these topics have also exhibited clustering. This interesting result indicates that sports shoes, as well as sportswear and equipment, are now tending towards mass customization, fashion, and personalization based on professional or competitive needs. After detailed analysis, the following aspects can explain the reasons for this result. (1) Empowerment by design. With the aid of AI and other design tools, the fashion industry’s development of sports shoe styles has been increasingly strengthened, making sports shoes an essential component of fashionable coordination (Mohiuddin Babu et al., 2022). (2) Change in lifestyle. Influenced by the social context, modern lifestyle has changed, leading to the popularization of casual and sportswear in daily life. Consumers are paying more attention to the coordination and fashion sense of clothing. The comfort and appearance of everyday wear may be more important than professional sports performance. Sports shoes are not just functional products; they also represent a lifestyle and set of values. Consumers may develop a sense of social belonging due to the lifestyle that sports shoes represent, such as sports, health and vitality. (3) Market positioning. Many sports shoe brands are expanding their target market from professional athletes to a broader consumer group, leading to product designs that focus more on the use of fashionable elements in different cultural markets. (4) Personalized expression. Most sports shoes on the market have met basic performance needs, so consumers start seeking differentiation in other aspects, such as design and personalization, to showcase personal style through apparel coordination (Zou et al., 2022b).

It is worth noting that although some topics are categorized as an Indifferent attribute, the importance users attribute to various elements of products and services varies, and the assessment of the importance of these needs should not be overlooked. Among the indifferent attributes, those with a high better coefficient, such as No. 8 Interactive display of material, No. 14 Animation and No. 27 sports community information which are topics with a high better coefficient. When a company’s financial resources and human capital are limited, it can selectively provide such services. However, it should be noted that these types of needs may also shift over time to become a Performance attribute or an Attractive attribute. Companies can continuously monitor their dynamics to make timely adjustments in decision-making, while appropriately reducing investment in needs that are deemed irrelevant. Interestingly, under the theme of “Spokesperson”, both No. 29 Avatar and No. 30 Real person have been identified as an Indifferent attribute. Avatar, as a very popular topic frequently discussed in recent years, but it is indeed an indifferent quality in the consumer shopping process. The reason for this phenomenon may be that consumers’ shopping habits typically focus on the actual value, price, quality, and functionality of the product (Venkatesh et al., 2022). Unless the avatar image is closely related to the product features, it may not become a primary factor in consumer decision-making.

## 5. Discussion

The results indicate that with the advent of the digital era represented by the metaverse, consumers’ online shopping experience has evolved from a focus on simple interface esthetics and functional implementation to a comprehensive experience centered on product functional attributes and brand value that addresses user emotions and personalized needs. Drawing on the collective research and the systematic analysis presented, this study proposes that in the new digital technology-enabled and modern context, the online shopping experience for sports shoes is augmented in four dimensions: platform experience augmentation, product experience augmentation, user demand augmentation, and interactive experience augmentation. These four dimensions together form the overall experience design (shown in Table 8).

**Figure 10:**
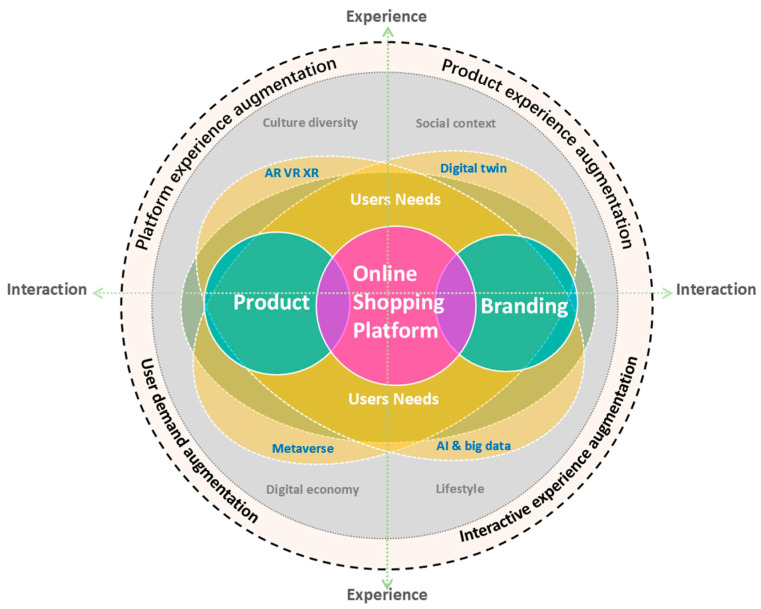
Innovative model for online retail experience.

The research findings reveal the following four augmentation dimensions of the online retail experience. Platform experience augmentation: By optimizing web design, improving operational fluidity, and enhancing the clarity of information layout, users can quickly and intuitively access the information they need, reducing cognitive load and enhancing the overall experience of using the platform. Product experience augmentation: Through the use of advanced technological display methods (such as VR and AR) and personalized customization services, more detailed product information is provided, helping users better understand the functions and features of products, thereby boosting purchasing confidence. User needs augmentation: Beyond focusing solely on product functionality, emphasis is placed on addressing users’ personalized needs, offering diverse services such as emotional support and care. Interactive experience augmentation: By incorporating advanced interactive technologies (such as virtual try-ons and personalized recommendations), the interactive experience between users and the platform is enhanced, increasing the sense of participation and immersion in the shopping experience. Through these four dimensions of enhancement, user demands for online shopping platforms are trending towards humanization, usability, emotionality, and technologization. This study constructs an innovative model for online retail experience (as shown in Figure 10). In this model, by innovatively integrating the various attributes of the products sold and the brand value culture, the design and development of online shopping platforms require the use of a variety of digital technologies. This connects the two major elements of product and brand, and through in-depth understanding and meeting the diverse needs of users, it promotes a high level of pleasure and participation in the interaction between consumers and online retail platforms. In this process, digital twins, AR, VR, metaverse, big data, artificial intelligence and other digital technologies can dynamically meet user needs, helping them to comprehensively understand the attributes of sports products and the value culture of related brands through the online shopping platform. Users, through continuous interaction with the online platform, can fully understand the product and brand, and achieve an online shopping user experience with high levels of usability, emotional and personal engagement, and technological interaction. This constructs an integrated experience design enhancement theory model that augments platform experience, product experience, user demand, and interactive experience.

This model can assist sports fashion brands and designers in related fields to better understand how to effectively utilize frontier digital technologies to construct the information architecture, display content, and interface interaction methods of online retail platforms, thereby gaining insights into consumers’ new demands for sports fashion products and providing a target system and design principles for future product design innovation and the optimization of online shopping platform design.

## 6. Conclusions

(1) Theoretical Implications

By mining comment themes and related feature words from authentic online posts and reviews of the online purchase experience of sports shoes and through the part-of-speech analysis of feature words, this paper constructs themes of user demand elements and integrates the Kano model and customer satisfaction indices to categorize user needs and determine the priority of supply for various indicators.

This study contributes to the research on online consumer experience needs by examining the consumer’s online shopping experience. This study explores a variety of factors that affect the experience, including the added value of products and services, interactive design elements, visual presentation (AR/VR presentation, virtual try-on, personalized shopping experiences, related scenario recommendations, dress way suggestions, etc.) and other highly interactive features based on cutting-edge digital technologies and their impact on consumer experience.

This study offers a new perspective on the product design and online service design for sports shoes business and constructs an innovation perspective and design guideline for sports shoe products and online service platforms. These design principles can be transferred to the online sales of other products and brand innovation processes based on the metaverse, informing knowledge production in design innovation.

(2) Managerial contributions

This study indicates that consumers respond positively to platforms that provide comprehensive sports shoe information, including size charts, product specifications, and customer reviews. Retailers should ensure that their platforms integrate detailed and accurate product descriptions, high-quality images, and user reviews to effectively address consumer concerns. The platforms should also incorporate interactive features such as virtual try-on and size guides to enhance the decision-making process. These features not only meet consumer needs but also bolster purchase confidence and reduce return rates. Furthermore, it is recommended that platforms continuously update and refine product information based on consumer feedback to maintain relevance and accuracy.

Secondly, the design of digital services based on advanced visual and interactive elements is crucial for enhancing users’ online shopping experience and satisfaction. Consumers are more inclined to utilize platform services that can provide an immersive visual shopping experience. Retailers should invest in high-quality visual effects, including 360-degree perspectives and detailed product close-ups. Offering features such as AR for virtual try-on allows consumers to better visualize how sports shoes will look and fit in real life, thereby significantly increasing their satisfaction. Furthermore, platforms should ensure that these interactive tools are user-friendly and seamlessly integrated with the shopping experience to maximize their effectiveness.

(3) Limitations and further research perspectives

While this study has achieved its research objectives to a certain extent, there are still limitations. The study explored consumers’ experiential needs for online sports shoe retail platforms and their underlying mechanisms; however, there has been no in-depth comparison and summary of the laws governing its development and changes. Future research could further substantiate these findings with longitudinal data from various online retail platforms. Employing panel data could provide a more comprehensive understanding of how consumer experience demands evolve over time.

Although the study offers insights into the general factors affecting consumer experience, it does not account for individual differences among consumers, such as income levels, age structures, shopping habits, brand loyalty, or sociodemographic characteristics. These factors could significantly influence consumer expectations and satisfaction. Future research should use methods such as fsQCA (Fuzzy-Set Qualitative Comparative Analysis) to investigate these individual differences, analyzing how various consumer characteristics interact with platform functionalities to affect the overall experience, thereby guiding future trends in the design and development of sports equipment.

In summary, while this study provides insights into consumers’ experiential needs for online sports shoe retail platforms, it does not provide practical and technical solutions for all research findings. Future research can conduct in-depth technical testing based on the research directions of this paper. It is believed that there will be more groundbreaking explorations in the near future.

## Figures and Tables

**Table 1:** Examples of related feature words.

Cluster	Topic	Number of Texts	Feature Words	Thematic Intensity
Topic 1	Platform or webpage	3886	term, item, guide, channel, platform, store, online, website, mall, computer, Taobao, shop, Jingdong, offline, WhatsApp, Lazada, phone, Tmall, Xiaomi	0.19413
Topic 2	Brand	2886	brand, commerce, company, industry, level, business, story, version, Nike, lining, Jordan, Tape, Converse, Anta, upper, board, classic, professional, premium, version	0.14418
Topic 3	Product Information	1577	type, data, technology, style, size, copyright, logo, carbon, sol, rubber, plastic, leather, silk, lace, steel, foam, fluorescent, black, white, gold, gray, peach, green, silver, blue, orange, brown, beige, pink, purple, colorful	0.078783
Topic 4	Service	2637	technology, interface, voice, feel, online, offline, screen, feel, feeling, sound, picture, visual, photo, text, video, animation, voice, feel, sense, dynamic	0.13174
Topic 5	Place	1700	Hangzhou, shop, Chinese, company, China, American, product, mall, local, domestic, Chicago, Singapore, London, Paris, Shanghai, Yangzhou, Putian, branch, location, vipshop	0.084928
Topic 6	Additional Service	1564	photo, grass, land, blossom, peach, growing, quality, Lining, black, size, white, standard, force, bos, price, industrial, power, offline, background, skin	0.078134
Topic 7	Marketing mode	3282	game, mail, topic, Karna, competition, link, article, story, post, love, heart, trend, trust, popular, original, professional, effective, genuine, positive, future, online, offline, official, college, street, land	0.16396
Topic 8	Stakeholders	2485	men, Chinese, woman, student, consumer, teacher, American, local, baby, girl, female, runner, adult, boy, British, designer, consumer, costumer, owner, manager, seller, buyer, player, supplier, charity, commission, agent, blogger	0.12414
Topic 9	Feedback of product or brand	10498	cheap, comfortable, real, specialty, crazy, congratulation, rich, lucky, cheaper, gorgeous, special, soft, dynamic, durable, authentic, normal, ideal, happy, negative, awesome, great, amazing, excellent, exclusive, cool	0.43573
Topic 10	Community	8931	post, share, community, club, blog, parent, team, men, woman, sister, girl, female, baby, chief, family, grandma, runner, city, run, platform, store, commerce, company, system	0.37069
Topic 11	Lifestyle	4664	shopping, classic, product, team, experience, woman, fashion, shop, technology, food, income, massage, Official, football, running, sport, game, college, street, dress code, training, decathlon, walk, bike, badminton, fish, runway, esthetic, road, land, peak, dance, pavilion, field	0.19358

**Table 2:** Coding and categorization of three-level indicators.

First-Level Indicators (Topic Information from LDA Modeling Analysis)	Second-Level Indicators	Third-Level Indicator (Feature Words from LDA Modeling Analysis)
Platform and Webpage	Webpage information and layout	term, item, guide, channel
	Retailing platform	platform, store, online, website, mall, computer, Taobao, shop, Jingdong, offline, WhatsApp, Lazada, phone, Tmall, Xiaomi
Brand	Business	brand, commerce, company, industry, level, business
	Brand name	Nike, lining, Jordan, Tape, Converse, Anta
	Brand positioning	upper, board, classic, professional, premium, version
	Market places	Chicago, Singapore, London, Paris, Shanghai, Yangzhou, Putian
	Stores and branch information	branch, location, vipshop
Product Information	Products categories	shoe, shoes, sneaker, shell, standard
	Basic information	type, data, technology, style, size, copyright, logo
	Material/fabric	carbon, sol, rubber, plastic, leather, silk, lace, steel, foam, fluorescent
	Price	discount, cost, sale, expensive, wholesale, free, sell, voucher, charge
	Color	black, white, gold, gray, peach, green, silver, blue, orange, brown, beige, pink, purple, colorful
	Details	sole, size, model, physical, insole, cushion, heel, cell, hole, midsole, sharp
	Product positioning/design positioning	upper, board, classic, fashion, dynamic, original, professional, level, effective, genuine, trend, specialty, usual, collection, concept, fashion
	Product features/advantages	function, weight, power, hard, matching, physical, balance, comfortable, soft, breathable, lightweight, quality, feel, technology,
	Design process	process, concept
	Making process	process, method
	Product data	data, rate, quality
Services	Service items	business, system, service, item, private
	Shopping process	gift, package, list, bill, payment, answer
	Appointment	book
	Delivery	ship, delivery
	After-sale	return, exchange, suggestion, policy, law, track
	Attitude	passion, professional, attitude, positive
Additional Services	Interactive or display methods	technology, interface, voice, feel, online, offline, screen, feel, feeling, sound, picture, visual, photo, text, video, animation, voice, feel, sense, dynamic
	Sports categories	sport, football, running, training, decathlon, ball, walk, bike, badminton, fish, dance, basketball
	Events	ticket, competition, program, club
	Dressing scenario	official, football, running, sport, game, college, street, dress code, training, decathlon, walk, bike, badminton, fish, runway, esthetic, road, land, peak, dance, pavilion, field
	Dressing scenario (Group)	parent, team, men, woman, sister, girl, female, baby, chief, family, grandma, runner, city, run
	Dressing time	time, morning, summer, day, hour, month, timely
	Relevant clothes and matching	slipper, pant, bag, watch, sandal, cloth, shirt, jean, clothes, sock
Marketing	Marketing mode	game, mail, topic, Karna, competition, link, article, story, post
	Branding content and brand identity	love, heart, trend, trust, popular, original, professional, effective, genuine, positive
	Marketing scenario	future, online, offline, official, college, street, land, competition
Stakeholders	Target consumer segment	men, Chinese, woman, student, consumer, teacher, American, local, baby, girl, female, runner, adult, boy, British
	Primary stakeholders	designer, consumer, costumer, owner, manager, seller, buyer, player, supplier
	Secondary stakeholders	charity, commission, agent, blogger
	People	men, woman, sister, girl, female, baby, chief, family, grandma, runner, city, run
	Spokesperson	Owen, star, team, Bos, avatar
Lifestyle	Lifestyle	food, income, massage, official, football, running, sport, game, college, street, dress code, training, decathlon, walk, bike, badminton, fish, runway, esthetic, road, land, peak, dance, pavilion, field
Community	Community	post, share, community, club, blog
Feedback of Product or Brand	Description of product or brand (positive)	cheap, comfortable, real, specialty, crazy, congratulation, rich, lucky, cheaper, gorgeous, special, soft, dynamic, durable, authentic, normal, ideal, happy, negative, awesome, great, amazing, excellent, exclusive, cool
	Feedback of product or brand (negative)	fake, shame, force, pain, stinky, false, upset

**Table 3:** Specific content of the questionnaire.

First-Level Indicators (Topic Information from LDA Modeling Analysis)	Second-Level Indicators	Third-Level Indicator (Feature Words from LDA Modeling Analysis)
Platform and Webpage	Webpage information and layout	term, item, guide, channel
	Retailing platform	platform, store, online, website, mall, computer, Taobao, shop, Jingdong, offline, WhatsApp, Lazada, phone, Tmall, Xiaomi
Brand	Business	brand, commerce, company, industry, level, business
	Brands name	Nike, Lining, Jordan, Tape, Converse, Anta
	Brand positioning	upper, board, classic, professional, premium, version
	Market places	Chicago, Singapore, London, Paris, Shanghai, Yangzhou, Putian
	Stores and branch information	branch, location, vipshop
Product Information	Products categories	shoe, shoes, sneaker, shell, standard
	Basic information	type, data, technology, style, size, copyright, logo
	Material /fabric	carbon, sol, rubber, plastic, leather, silk, lace, steel, foam, fluorescent
	Price	discount, cost, sale, expensive, wholesale, free, sell, voucher, charge
	Color	black, white, gold, gray, peach, green, silver, blue, orange, brown, beige, pink, purple, colorful
	Details	sole, size, model, physical, insole, cushion, heel, cell, hole, midsole, sharp
	Product positioning/design positioning	upper, board, classic, fashion, dynamic, original, professional, level, effective, genuine, trend, specialty, usual, collection, concept, fashion
	Product features/advantages	function, weight, power, hard, matching, physical, balance, comfortable, soft, breathable, lightweight, quality, feel, technology,
	Design process	process, concept
	Making process	process, method
	Product data	data, rate, quality
Services	Service items	business, system, service, item, private
	Shopping process	gift, package, list, bill, payment, answer
	Appointment	book
	Delivery	ship, delivery
	After-sale	return, exchange, suggestion, policy, law, track
	Attitude	passion, professional, attitude, positive
Additional Services	Interactive or display methods	technology, interface, voice, feel, online, offline, screen, feel, feeling, sound, picture, visual, photo, text, video, animation, voice, feel, sense, dynamic
	Sports categories	sport, football, running, training, decathlon, ball, walk, bike, badminton, fish, dance, basketball
	Events	ticket, competition, program, club
	Dressing scenario	official, football, running, sport, game, college, street, dress code, training, decathlon, walk, bike, badminton, fish, runway, esthetic, road, land, peak, dance, pavilion, field
	Dressing scenario (Group)	parent, team, men, woman, sister, girl, female, baby, chief, family, grandma, runner, city, run
	Dressing time	time, morning, summer, day, hour, month, timely
	Relevant clothes and matching	slipper, pant, bag, watch, sandal, cloth, shirt, jean, clothes, sock
Marketing	Marketing mode	game, mail, topic, Karna, competition, link, article, story, post
	Branding content and brand identity	love, heart, trend, trust, popular, original, professional, effective, genuine, positive
	Marketing scenario	future, online, offline, official, college, street, land, competition
Stakeholders	Target consumer segment	men, Chinese, woman, student, consumer, teacher, American, local, baby, girl, female, runner, adult, boy, British
	Primary stakeholders	designer, consumer, costumer, owner, manager, seller, buyer, player, supplier
	Secondary stakeholders	charity, commission, agent, blogger
	People	men, woman, sister, girl, female, baby, chief, family, grandma, runner, city, run
	Spokesperson	Owen, star, team, Bos, avatar
Lifestyle	Lifestyle	food, income, massage, official, football, running, sport, game, college, street, dress code, training, decathlon, walk, bike, badminton, fish, runway, esthetic, road, land, peak, dance, pavilion, field
Community	Community	post, share, community, club, blog
Feedback of Product or Brand	Description of product or brand (positive)	cheap, comfortable, real, specialty, crazy, congratulation, rich, lucky, cheaper, gorgeous, special, soft, dynamic, durable, authentic, normal, ideal, happy, negative, awesome, great, amazing, excellent, exclusive, cool
	Feedback of product or brand (negative)	fake, shame, force, pain, stinky, false, upset

**Table 4:** Kano questionnaire content.

No.	Themes	Topics	Detailed Descriptions
1	Platform and Webpage Design	Beautiful interface	Reasonable design of fonts, colors, graphics, etc.
2		Smooth operation	Improve the efficiency of one-handed interaction
3		Clear layout	Reasonable layout of product categories, functional areas and other items, easily accessible information
4	Product Information	Immediate information	Timely update of information
5		Product basic information	Graphical presentation of sports shoes basic information (including color, price, style, etc.)
6		Product special features information	Graphic introduction to the design, style, fabric, etc., special features for the sports shoes
7	Interactive Display	Interactive display of fabrics	Visualization of fabrics used in sports shoes
8		Interactive display of material	Visualization of special materials used in sports shoes
9		Interactive display of high-performance features	Visualization of waterproof, breathable, high elasticity and ultra-light performance of sports shoes
10		Interactive display of comprehensive product features	A comprehensive display of sports shoe design, esthetics, shape, fabrics, materials, features and functions
11		Interactive display of making techniques	Visualization of making techniques, cutting techniques, sewing techniques, etc., of sports shoes
12		Interactive display of package	Visualization of package
13	Interactive Display Methods	VR and AR	The use of VR and AR technologies in interactive display
14		Animation	Use animation to present information in online shopping platforms
15		Video	The use of new media as a way of presenting sports shoe product information on online shopping platforms
16		Sound	Provide the background music in online shopping platforms
17		Touch	Providing tactile perception of the fabric or material of sports shoes
18	Additional Services	After-sales service policy	Provide relevant after-sales service policy information
19		Platform user evaluation	Should online shopping platforms provide user reviews and rating systems for shopping experiences?
20		Size testing	If online size testing function is provided
21		Personalized customization	Providing personalized and customized options for sports shoes products on online shopping platforms
22		Virtual try-on	Providing immersive virtual try-on based on individual image in online shopping platforms
23		Dressing way recommendations	Providing coordination schemes for related apparel such as clothing, accessories, etc., for sports shoes
24		Dress scenario recommendations	Providing and visualization presentation of this sportswear scenario (sports scene or sports occasion) recommendations
25		Product usage data	Providing data on the usage duration, wear status of sports shoes, and related visual presentations
26	Community	Product community information	Provide community platform information for product communication and sharing
27		Sports community information	Provide community platform information for sports events or sports activities
28	Feedback	Product comment	Provide information about product reviews from customers who have purchased this product
29	Spokesperson	Avatar	If the product spokesperson image in an online shopping platform is a digital avatar
30		Real people	If the product spokesperson image in the online shopping platform is a real person? (e.g., sports stars, entertainment icons, community figures, designers, etc.)

**Table 5:** Kano standardization rules.

Function/Service	Negative Questions					
		Highly Dislike (1 Score)	Acceptable (2 Scores)	Indifferent (3 Scores)	As Expected (4 Scores)	Highly Like (5 Scores)
Positive questions	Highy Dislike (1 score)	Q	R	R	R	R
	Acceptable (2 scores)	M	I	I	I	R
	Indifferent (3 scores)	O	I	I	I	R
	As Expected (4 scores)	M	I	I	I	R
	Highly like (5 scores)	Q	A	A	A	Q

Notes: A: Attractive attribute, O: Performance attribute, M: Must-be attribute, I: Indifferent attribute, R: Reverse attribute, Q: Questionable attribute.

**Table 6:** Statistical results of the Kano survey (the unit of the value is percentage).

No.	Topic	M	O	A	I	R	Q	C	Better	Worse
1	Beautiful interface	40.00	25.70	22.60	11.70	0.00	0.00	M	48.30	−65.70
2	Smooth operation	50.18	18.12	24.47	7.23	0.00	0.00	M	42.59	−68.30
3	Clear layout	43.43	16.17	26.73	13.67	0.00	0.00	M	42.90	−70.23
4	Immediate information	12.91	44.17	15.53	20.19	5.63	1.55	O	64.33	−61.51
5	Product basic information	36.07	30.33	7.38	18.85	0.00	7.38	M	40.71	−71.68
6	Product special features information	11.36	49.81	10.19	22.72	5.44	0.49	O	63.78	−65.02
7	Interactive display of fabrics	5.44	9.42	28.83	47.09	8.74	0.49	I	42.14	−16.36
8	Interactive display of material	2.46	18.85	33.61	36.89	0.00	8.20	I	52.46	−21.31
9	Interactive display of high-performance features	5.92	5.83	34.08	45.15	8.93	0.10	I	43.86	−12.91
10	Interactive display of comprehensive product features	22.72	35.44	23.88	12.33	3.79	1.84	O	62.86	−61.63
11	Interactive display of making techniques	18.35	40.39	10.49	22.52	7.09	1.17	O	55.45	−64.02
12	Interactive display of package	6.50	7.18	33.30	45.15	7.67	0.19	I	43.94	−14.86
13	VR and AR	6.21	11.55	48.16	27.38	6.12	0.58	A	64.00	−19.04
14	Animation	2.46	18.85	31.15	36.07	0.00	11.48	I	56.48	−24.07
15	Video	4.37	14.56	42.23	33.4	2.72	2.72	A	60.06	−20.02
16	Sound	0.82	4.10	18.03	64.75	4.10	8.20	I	25.23	−5.61
17	Touch	5.83	16.02	43.88	30.49	3.30	0.49	A	62.26	−22.7
18	After-sales service policy	26.12	31.94	25.44	12.04	1.26	3.20	O	60.06	−60.77
19	Platform user evaluation	3.40	9.51	33.5	46.02	6.12	1.46	I	46.53	−13.97
20	Size testing	18.93	40.29	20.00	16.80	2.33	1.65	O	62.79	−61.68
21	Personalized customization	6.99	12.82	45.92	28.83	3.69	1.75	A	62.11	−20.94
22	Virtual try-on	3.50	18.45	41.17	33.79	2.62	0.49	A	61.52	−22.65
23	Dressing way recommendations	8.93	12.23	48.16	27.18	2.33	1.17	A	62.58	−21.93
24	Dress scenario recommendations	5.24	18.83	41.17	32.33	2.23	0.19	A	61.49	−24.68
25	Product usage data	3.79	9.32	31.65	47.57	6.31	1.36	I	44.37	−14.20
26	Product community information	6.99	6.02	35.24	45.05	6.21	0.49	I	44.22	−13.94
27	Sports community information	3.20	10.49	36.12	41.94	6.21	2.04	I	50.79	−14.92
28	Product comment	19.80	41.16	21.00	15.90	2.0	0.14	O	63.52	−62.29
29	Avatar	4.27	9.13	34.17	45.53	5.63	1.26	I	46.51	−14.39
30	Real people	7.77	5.53	35.92	42.04	8.64	0.10	I	45.43	−14.57

**Table 7:** Summary of important topics’ attributes.

Attribute	Themes	Topics	Features
Must-be attribute (M)	Webpage design	No. 1 Beautiful interface	Usability, esthetics, accessibility
		No. 2 Smooth operation	
		No. 3 Clear layout	
	Product information	No. 5 Product basic information	Basic product information
Performance attribute (O)	Product information	No. 4 Immediate information	Immediacy
		No. 6 Product special features information	Products value-added, personalization
	Interactive display	No. 10 Interactive display of comprehensive product features	Interactivity
		No. 11 Interactive display of making techniques	Interactivity, safety, technologization
	Additional services	No. 18 After-sales service policy	Interactivity, safety, technologization
		No. 20 Size testing	Safety, immersion, experiential, interactivity
	Feedback	No. 28 Product comment	Safety
Attractive attribute (A)	Interactive display methods	No. 13 VR and AR	Socialization, interactivity, technologization
		No. 15 Video	
		No. 17 Touch	
	Additional services	No. 21 Personalized customization	Personalization
		No. 22 Virtual try-on	Immersion, experiential
		No. 23 Dressing way recommendations	Personalization, socialization
		No. 24 Dress scenario recommendations	Personalization, socialization, belonging

**Table 8:** Overall experience influences.

Augmentation Dimensions	Experience Needs	
Platform experience augmentation	Usability, esthetics, accessibility, immediacy	Overall experience: humanization, usability, emotionality, technologization
Product experience augmentation	Tangible products and intangible products (service); Economy, structure, function, technology, esthetics. Products and services value-added enabled by technology.	
User demand augmentation	Safety, utility, comfort; identity, emotional support, belonging, personalization	
Interactive experience augmentation	Interactivity, personalization, socialization, technologization, immersion	

## Data Availability

Data are contained within the article.
